# Multiple-Herbicide Resistance Is Widespread in Roadside Palmer Amaranth Populations

**DOI:** 10.1371/journal.pone.0148748

**Published:** 2016-04-12

**Authors:** Muthukumar V. Bagavathiannan, Jason K. Norsworthy

**Affiliations:** 1 Department of Soil and Crop Sciences, Texas A&M University, College Station, Texas, United States of America; 2 Department of Crop, Soil, and Environmental Sciences, University of Arkansas, Fayetteville, Arkansas, United States of America; Instituto de Agricultura Sostenible (CSIC), SPAIN

## Abstract

Herbicide-resistant Palmer amaranth is a widespread issue in row-crop production in the Midsouthern US. Palmer amaranth is commonly found on roadside habitats in this region, but little is known on the degree of herbicide resistance in these populations. Herbicide resistance in roadside Palmer amaranth populations can represent the spread of an adaptive trait across a selective landscape. A large-scale survey was carried out in the Mississippi Delta region of eastern Arkansas to document the level of resistance in roadside Palmer amaranth populations to pyrithiobac and glyphosate, two important herbicides with broad history of use in the region. A total of 215 Palmer amaranth populations collected across 500 random survey sites were used in the evaluations. About 89 and 73% of the surveyed populations showed >90% survival to pyrithiobac and glyphosate, respectively. Further, only 3% of the populations were completely susceptible to glyphosate, while none of the populations was completely controlled by pyrithiobac. Among the 215 populations evaluated, 209 populations showed multiple resistance to both pyrithiobac and glyphosate at varying degrees. Dose-response assays confirmed the presence of high levels of herbicide resistance in the five selected populations (≥ 25-fold compared to a susceptible standard). Results demonstrate the prevalence of multiple-herbicide resistance in roadside Palmer amaranth populations in this region. Growers should be vigilant of Palmer amaranth infestation in roadsides adjacent to their fields and implement appropriate control measures to prevent likely spread of herbicide resistance into their fields.

## Introduction

Glyphosate has been the major weed management tool in glyphosate-resistant crops, leading to the widespread evolution of glyphosate-resistant weeds, particularly in the southern US. Palmer amaranth (*Amaranthus palmeri*) is currently a widespread glyphosate-resistant weed issue in row-crop production in the southern US [1,2]. A native of Southwestern US deserts [3], this species has spread to arable fields, with a potential for adapting to different environments. Until the widespread adoption of glyphosate-resistant crops, acetolactate synthase (ALS) inhibitors were heavily relied upon for the management of Palmer amaranth in row crops in the southern region. The first ALS-inhibitor-resistant Palmer amaranth (resistance to imidazolinones) in the US was documented in Kansas in 1993 [4]. Subsequently in 1994, ALS-inhibitor resistance (resistance to sulfonylureas, imidazolinones, and pyrimidinyl(thio)benzoate) was documented in Arkansas [5] and Tennessee [5] (resistance to imidazolinones and pyrimidinyl(thio)benzoate). ALS-inhibitor resistance was starting to be widespread in the Midsouthern region by the mid to late 90’s [6]. However, the arrival of glyphosate-resistant crops provided an effective means for management of ALS-inhibitor-resistant Palmer amaranth in this region.

The first glyphosate-resistant Palmer amaranth population was discovered in 2004 in a field in Macon County, Georgia [7]. This population exhibited >12-fold resistance to glyphosate compared to a susceptible standard. A year later, glyphosate-resistant Palmer amaranth was found in Arkansas [8]. Soon thereafter, this species had rapidly spread to other areas in the Midsouthern US and also to other parts of the country, including the Southwestern [9], Midwestern [10] and Northcentral [11] states. Palmer amaranth is currently infesting crop lands in at least 24 US states [5]. Some populations also exhibit multiple resistance to both ALS-inhibitors and glyphosate in the southern US [12]. Palmer amaranth is widely considered an invasive, high consequence pest, and its ability for rapid growth, high fecundity, high genetic diversity, tolerance to adverse conditions, and high tendency for evolving herbicide resistance have enabled the dominance of Palmer amaranth in agricultural systems [13].

**Fig 1:**
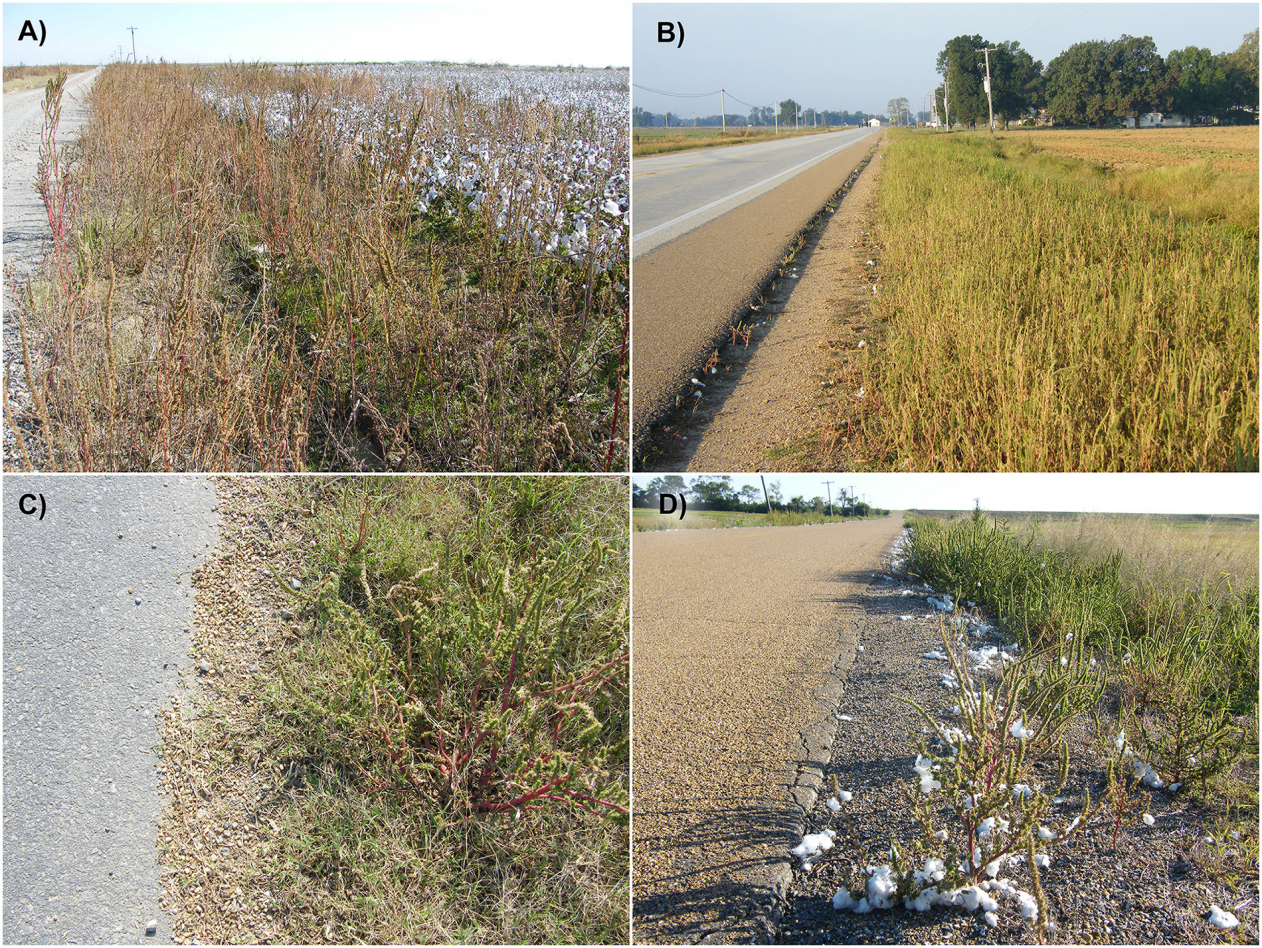
Images illustrating the occurrence of Palmer amaranth on roadside habitats in eastern Arkansas. A) along a rural road adjacent to a cotton production field, B) along a state highway, C) among grain spill on road shoulders, and D) among cotton lint spill on road shoulders.

In addition to arable crop production fields, Palmer amaranth populations are also found in roadsides and other natural areas in the Midsouthern US (Fig 1A–1D). A recent survey conducted in the eastern Arkansas region revealed that Palmer amaranth was the predominant arable weed, occurring in 64% of the survey sites [14]. It is important to note that Palmer amaranth was not usually found in roadsides prior to the widespread evolution of glyphosate-resistance in this species (Ken Smith and John Boyd, Pers. Comm.). It is believed that heavy infestations of glyphosate-resistant Palmer amaranth in production fields has favored its dispersal to roadside habitats. Anecdotal evidence suggests that transport of agricultural commodities to nearby elevators and movement of farm machineries across fields greatly contribute to the dispersal of Palmer amaranth to roadside habitats away from the infested fields [15]. Field observations also suggest that the roadside Palmer amaranth populations establish self-sustaining populations through establishment of a persistent soil seedbank (authors’ personal observations). While agricultural operations greatly contribute to the dispersal of Palmer amaranth from crop fields to roadsides, the roadsides themselves can facilitate further spread of Palmer amaranth populations for much longer distances. The importance of roads as corridors for the spread of invasive plant species has been well-established [16–18]. In particular, vehicles can move weed seeds for long distances [19].

The occurrence of herbicide resistance in roadside Palmer amaranth populations can have implications for the management of herbicide resistance in production fields. However, little is known on the resistance status of roadside Palmer amaranth populations occurring in the Mississippi Delta region, one of the most important agricultural production regions in the Midsouthern US. The objective of this study is to document the occurrence of Palmer amaranth resistance to pyrithiobac and glyphosate in a large collection of roadside Palmer amaranth populations from eastern Arkansas.

## Materials and Methods

### Survey and sample collection

A landscape-scale survey was conducted in fall 2012 across the Mississippi Delta region in eastern Arkansas, spanning from the Louisiana border in the south (N33° 01’ 29”; W91° 12’ 17”) to Missouri border in the north (N35° 59’ 20”; W89° 56’ 56”). The survey included 500 roadside sites in this region. The survey sites were pre-determined following a stratified random survey methodology without any specific knowledge of the locations, but included different road types such as interstate highways, state highways, gravel roads, and dirt roads. The sites were chosen such that they were at least 5 km apart from each other. More details on the survey methodology is described in Korres et al [14]. Site and landscape characteristics, including road type, vegetation cover, slope, ditch width, and nearby land-use pattern were also recorded at each survey site (see Korres et al [14]).

Palmer amaranth inflorescences were collected from the survey sites where the species was present, and samples from a total of 215 randomly selected populations were used in the present study. In each site, inflorescences were harvested from 15 random plants (1 per female plant) and placed in a paper bag. No specific permissions were required for these locations/activities because the samples were collected from public lands that do not impose any restrictions on plant sample collection. Further, the field studies did not involve any endangered or protected species. The samples were brought to the laboratory, dried at 40 C for 72 hours and processed subsequently by grinding the entire inflorescence using a motorized mill. The seed material was stored in a cold room at 4 C until used for herbicide assays.

### Herbicide assays

The herbicide assays were carried out in the greenhouse between December 2012 and May 2013). Each study population was treated separately with 2X pyrithiobac (trade name: Staple^®^; 1X rate = 73 g ai ha^-1^) and 1X glyphosate (trade name: Roundup Weather Max^®^; 870 g ae ha^-1^). These rates were selected considering that Palmer amaranth is usually more sensitive to glyphosate than it is to pyrithiobac. A non-ionic surfactant (Induce^®^, Helena Chemical Co., Collierville, TN) was added to pyrithiobac applications at 0.25% volume/volume (v/v).

Seeds pertaining to each population were planted in plastic flats (6.5 cm x 40 cm x 54 cm) containing potting soil mix (LC1, SunGro Horticulture Canada Ltd). Treatments were applied to each flat and the experiment was repeated over time (two runs; first run between December 2012 and February 2013, second run between March 2013 and May 2013). At emergence, seedlings were thinned to achieve a density of about 100 seedlings in each flat. Thinning ensured that seedling densities were uniform and that there was adequate spray coverage. A known susceptible Palmer amaranth population (SC1986) was used as a susceptible standard in the assays. The SC1986 population was collected in 1986 from Clarendon County, South Carolina prior to herbicide resistance evolution in this species (see Norsworthy et al [8]). A susceptible population that has not been treated with pyrithiobac or glyphosate was not readily available from Arkansas. A nontreated standard was also maintained for comparison. Treatments were applied on 2- to 3-leaf seedlings, using an automated spray chamber calibrated to deliver 143 liters of spray solution ha^-1^ at a pressure of 276 kPa. Standard greenhouse conditions (16 hr photoperiod and 30/20 C day/night temperature) were maintained throughout the study, and the flats were watered as required.

Plant survival was documented 14 days after herbicide treatment (DAT). For each treatment, percent survival (out of total number of seedlings treated) and control ratings (overall control of the population compared to the nontreated standard) were recorded. Control ratings were on a scale of 0–100, with 0 representing no plant injury or growth reduction compared to the nontreated standard and 100 indicating complete plant death.

### Dose-response analysis

Following the herbicide screening, five populations that showed high survival were randomly selected for further confirming resistance using dose-response analysis (see Table 1 for details). The SC1986 population was used as the susceptible standard. The dose-response assay included eight doses for the resistant biotypes (1/8X, 1/4X, 1/2X, 1X, 2X, 4X, 8X and 16X) and the susceptible standard (1/128X, 1/64X, 1/32X, 1/16X, 1/8X, 1/4X, 1/2X and 1X). The field rates (1X) used were 73 g ai ha^-1^ for pyrithiobac and 870 g ae ha^-1^ for glyphosate. Applications of pyrithiobac also included a non-ionic surfactant at 0.25% v/v.

**Table 1:** Results of dose-response analysis of selected roadside Palmer amaranth populations to glyphosate.

Population	GPS coordinates	Log-logistic regression equation	RMS value^a^	LD_50_ value^b^	R/S ratio^c^
R57	N33° 12’ 46”; W91° 19’ 01”	Y = 100/ [exp(0.99(log(x)-log(1737)))]	60.2	1,737	25.0
R85	N33° 30’ 15”; W91° 18’ 10”	Y = 100/ [exp(0.70(log(x)-log(1101)))]	25.5	1,101	15.8
R162	N34° 08’ 26”; W91° 17’ 36”	Y = 100/ [exp(0.99(log(x)-log(310)))]	19.5	310	4.5
R289	N34° 54’ 14”; W90° 31’ 34”	Y = 100/ [exp(0.40(log(x)-log(640)))]	116.9	640	9.2
R416	N35° 35’ 22”; W90° 10’ 12”	Y = 100/ [exp(0.5(log(x)-log(1294)))]	68.9	1,294	18.6
SC1986 (S)^d^	Clarendon County, SC	Y = 100/ [exp(1.09(log(x)-log(69.5)))]	155.6	69.5	-

^a^ RMS indicates residual means square value for the fitted model

^b^LD_50_ value indicates the amount of active ingredient (g ai ha^-1^) required to cause 50% mortality in the test population

^c^R/S ratio indicates the ratio of LD50 value of the given biotype divided by the LD50 value of the susceptible standard

^d^SC1986 is the susceptible Palmer amaranth standard which was collected in 1986 from Clarendon County, South Carolina

Seeds from each chosen population were sown in flats as described above. At the 1-leaf stage, seedlings were transplanted to individual pots (15 cm-diameter) and allowed to establish. A total of 20 well-established seedlings were used for each dose. The treatments were applied at the 2- to 3-leaf stage, following the same procedure described above. Seedling survival was documented at 21 DAT and presented as percent survival (out of the 20 seedlings treated for each dose). The dose-response assay was also repeated over time.

### Data analysis

Data pertaining to percent survival and overall control ratings from the herbicide screening were presented using histograms to show the degree of Palmer amaranth resistance to pyrithiobac and glyphosate in surveyed populations. Differences between the two experimental runs were tested using the non-parametric NPAR1WAY produce in SAS (version 9.4, The SAS Institute, Cary, NC), following the Kuiper statistic. No significant differences were found between the runs for each herbicide treatment; thus, data were pooled between the two runs.

Data from the dose-response assay was fitted to a four-parameter log-logistic model using the non-linear regression (PROC NLIN) procedure of SAS, which took the following form:

$$\;Y=C+\frac{D-C}{1+\text{exp}\left[b\left(\text{log}\left(x\right)-\text{log}\left(LD_{50}\right)\right)\right]}$$

where *C* is the lower asymptote (constrained at 0), *D* is the upper asymptote (constrained at 100), *LD*_50_ is the dose yielding 50% mortality and *b* is the slope of the curve at *LD*_50_ Resistance ratio (R/S ratio) was calculated by dividing the *LD*_50_ value of the given resistant population by the *LD*_50_ value of the susceptible standard.

## Results and Discussion

**Fig 2:**
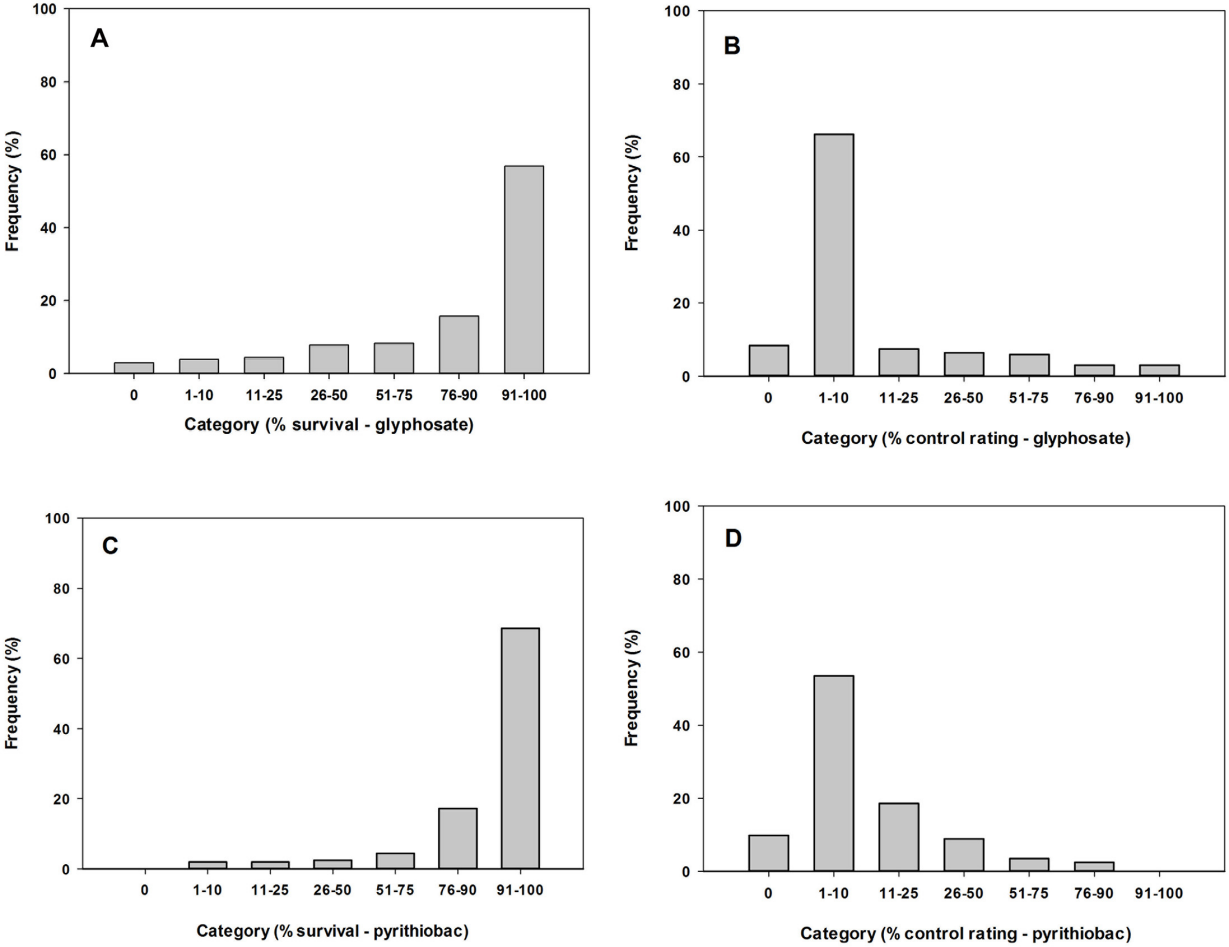
Histograms showing frequency distribution of the surveyed populations. A) survival (%) to 1X glyphosate, B) control ratings (%) to 1X glyphosate compared to a non-treated standard, C) survival (%) to 2X pyrithiobac, and D) control ratings (%) to 2X pyrithiobac compared to a non-treated standard. The field rates (1X) used were 73 g ai ha^-1^ for pyrithiobac and 870 g ae ha^-1^ for glyphosate. Applications of pyrithiobac also included a non-ionic surfactant at 0.25% v/v.

**Fig 3:**
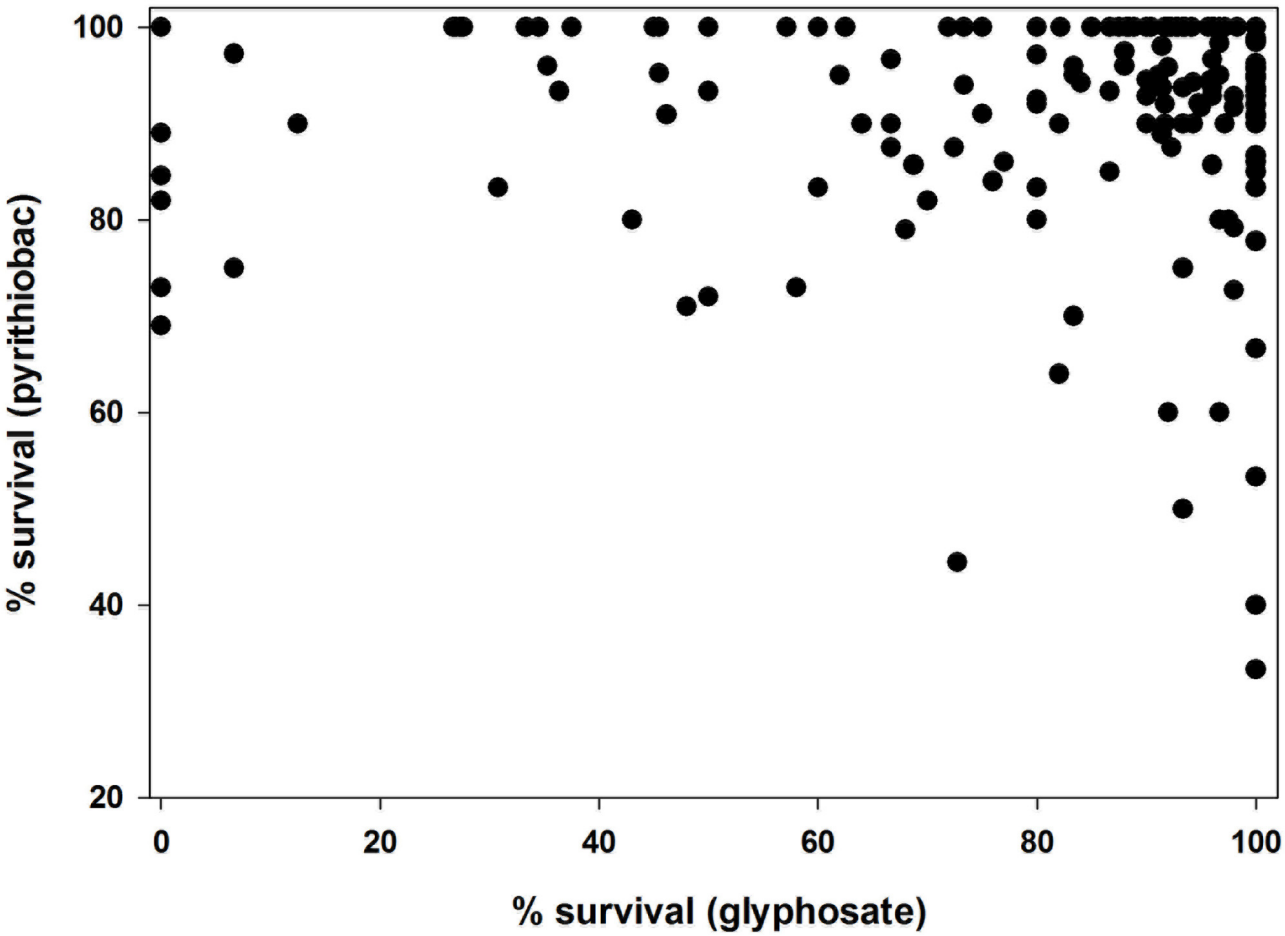
Multiple resistance profiles of surveyed roadside Palmer amaranth populations to glyphosate (X axis) and pyrithiobac (Y axis). Each data point represents the resistance status of each population for the two herbicides.

The study revealed a widespread occurrence of resistance to pyrithiobac and glyphosate in roadside Palmer amaranth populations. This is the first study to report the prevalence of herbicide resistance in an arable weed colonizing non-agricultural areas. Results showed that approximately 60 and 70% of the populations had >90% of the individuals within a population surviving to glyphosate (Fig 2A) and pyrithiobac (Fig 2C), respectively. Moreover, about 70 and 55% of the surveyed Palmer amaranth populations showed <10% control (based on injury and growth reduction) to glyphosate (Fig 2B) and pyrithiobac (Fig 2D), respectively. About 97% of the populations contained at least few survivors (>1% of treated plants) to glyphosate, while none of the populations was completely susceptible to pyrithiobac. Further, most of the surveyed populations had multiple resistance to both herbicides. Among the 215 populations evaluated, 209 populations showed multiple resistance at varying degrees (Fig 3).

Dose-response analyses confirmed the presence of high levels of resistance to glyphosate in the selected Palmer amaranth populations (Table 1). For pyrithiobac dose-response, curve fitting was not possible for any of the selected populations since the highest dose used (16X, 1,168 g ai ha^-1^) failed to cause >50% mortality. Based on the LD_50_ value of 44.2 g ai ha^-1^ for the susceptible standard (not shown), the selected Palmer amaranth populations were >26-fold resistant to pyrithiobac. For glyphosate, the highest resistant population (R57) exhibited a 25-fold resistance, with an LD_50_ value of 1,737 g ae ha^-1^ (Table 1). These observations are consistent with previous evaluations on resistant Palmer amaranth populations (field origin) for ALS-inhibitor herbicides [6] and glyphosate [7].

Confirmation of herbicide resistance in roadside Palmer amaranth populations presents a serious concern because these populations can facilitate further spread of resistance to adjacent crop production fields and non-cultivated areas. Herbicide resistance can spread to pristine populations in adjacent production fields through the movement of pollen, seed, and vegetative propagules from resistant plants occurring on the roadsides and vice versa. Even if resistant weeds are eliminated in the crop fields, propagule movement from roadside populations could contribute to re-establishment of resistance in cultivated fields. Given that roadside environment can favor the spread of plant species [16–18] and along with it herbicide resistance traits, there are high likelihoods for secondary dispersal of resistance alleles for much longer distances. In fact, resistant Palmer amaranth populations were found in roadside habitats in places far away from production fields, including residential areas, rail roads, abandoned sites, woodlands, and pasture lands [15], indicating the ubiquity of herbicide-resistant Palmer amaranth in the Mississippi Delta region.

For Palmer amaranth, landscape-level dispersal of resistance can occur through the movement of pollen and seed. Palmer amaranth is a dioeceous species (separate male and female plants), which favors outcrossing. Sosnoskie et al. [20] documented about 20 to 40% outcrossing in Palmer amaranth at 300 m from the pollen source and it was likely that low levels of gene flow could have occurred at much farther distances, which were not tested. Given this finding, the likelihood for exchange of resistance alleles between the roadside and adjacent field Palmer amaranth populations through pollen-mediated gene flow is very high. Our field observations suggest that commodity transport is an important vector for Palmer amaranth seed dispersal [15]. Seed spill occurs along the roadsides when commodities are transported in trucks from production fields to nearby grain handling facilities (Fig 1C). Palmer amaranth seeds can be carried by cotton lint and disperse during transport to nearby ginning mills (Fig 1D). Due to their small size, Palmer amaranth seeds are easily dispersed through machineries such as combine harvesters and tillage equipment [15].

Herbicide resistance in roadside Palmer amaranth populations could be pre-existing from the source of origin (original production field) and to some extent, resistance could have been acquired through pollen-mediated gene flow from adjacent fields infested with resistant Palmer amaranth. However, the evaluations showed high frequencies of resistant individuals within the majority of roadside populations, suggesting that resistance has been selected at the source of origin and that it was perhaps pre-existing before dispersal on the roadsides. Glyphosate or pyrithiobac are rarely sprayed as a blanket application on roadsides in the Mississippi Delta region, thus selection for resistance is less likely to occur on the roadsides. Irrespective of the source, the potential for Palmer amaranth to recruit and establish in roadside habitats implies that the roadside Palmer amaranth populations can serve as conduits for the spread of herbicide resistance to nearby production fields.

## Conclusion

Efforts to prevent and manage herbicide resistance should consider the role of roadside weed populations on the spread of herbicide resistance. Management of resistant weed populations in non-agricultural sites will require community efforts, a lack of which is a major hurdle for the implementation of effective resistance management programs at regional levels. An example of such efforts is the establishment of a ‘Zero Tolerance’ program for Palmer amaranth in Clay County, AR which involved collective efforts by growers, county agents, and extension personnel to eliminate Palmer amaranth from the region. Such efforts are vital for effectively preventing and managing herbicide resistance evolution. In this respect, government incentives can greatly encourage and support community efforts in herbicide resistance management.
